# Validation of a Prediction Model for Acute Kidney Injury after Cardiac Surgery in a Retrospective Asian Cohort

**DOI:** 10.3390/jcm13102740

**Published:** 2024-05-07

**Authors:** Pei-Hsin Tsai, Jun-Sing Wang, Ching-Hui Shen

**Affiliations:** 1 Department of Anesthesiology, Taichung Veterans General Hospital, Taichung 407219, Taiwan; tsaipeihsin1019@gmail.com; 2 Division of Endocrinology and Metabolism, Department of Internal Medicine, Taichung Veterans General Hospital, Taichung 407219, Taiwan; 3 Department of Post-Baccalaureate Medicine, College of Medicine, National Chung Hsing University, Taichung 402202, Taiwan; 4 Department of Medicine, School of Medicine, National Yang Ming Chiao Tung University, Taipei 112304, Taiwan

**Keywords:** acute kidney injury, cardiovascular surgery, chronic kidney disease, estimated glomerular filtration rate

## Abstract

**Background:** The incidence of postoperative acute kidney injury (AKI) is relatively high in some Asian regions. The objective of this study was to examine the performance of an AKI prediction model developed based on data from a White-dominant population in a retrospective Asian cohort of patients undergoing cardiovascular surgery. **Methods:** We retrospectively identified 549 patients who underwent elective major cardiovascular surgery (coronary artery bypass graft, valve surgery, and aorta surgery), and excluded those who underwent a percutaneous cardiovascular procedure. Patients with a baseline estimated glomerular filtration rate (eGFR) < 60 mL/min/1.73 m^2^ were also excluded. AKI was defined according to the Kidney Disease: Improving Global Outcomes (KDIGO) definition. Performance of the prediction model for AKI was expressed as area under the receiver operating characteristic curve (AUC). **Results:** The prediction model had a good predictive accuracy for postoperative AKI (all AUC > 0.92). The AUC of the prediction model in subgroups of age (<65 years and ≥65 years), sex (male and female), hypertension, and diabetes were all >0.85 (all *p* values < 0.001). **Conclusions:** The model could be used to predict postoperative AKI in Asian patients undergoing cardiovascular surgery with a baseline eGFR ≥ 60 mL/min/1.73 m^2^.

## 1. Introduction

Acute kidney injury (AKI), defined as a rapid decrease in renal function, is a common complication in patients admitted to hospitals. According to a large study in which more than 520,000 patients were investigated [1], the prevalence of in-hospital AKI was around 10–15%. Among patients who were admitted to an intensive care unit, the prevalence may be even higher than 55% [2]. In a multinational cross-sectional study [2], critically ill patients with AKI were associated with a longer length of stay in the intensive care unit (6 days vs. 4 days, *p* < 0.001) and in the hospital (15 days vs. 12 days, *p* < 0.001). Moreover, patients with AKI have been associated with adverse long-term outcomes, including development of chronic kidney disease (CKD) or end-stage kidney disease [3,4,5], and an increase in mortality [4,5]. In a population-based cohort study that included nearly 3800 patients with AKI who required in-hospital dialysis and survived free of dialysis for at least 30 days after discharge [3], the risk of chronic dialysis significantly increased (adjusted hazard ratio 3.23, 95% CI 2.70 to 3.86) compared with matched controls during a median follow-up period of 3 years.

Approximately one-third of AKI episodes in hospitalized patients occur in the postoperative setting [6,7]. In a recent large prospective international observational multi-center study [8], one in five patients had postoperative AKI after a major surgery. Furthermore, one in ten of these patients had AKI beyond 7 days after the surgery [9]. The authors observed that a postoperative AKI was associated with adverse outcomes, including length of hospital stay and mortality [8,9]. These findings were in line with those of a previous observational cohort study [10] in which postoperative AKI occurred in 11.8% of more than 160,000 major surgery hospitalizations investigated. It is not clear if this type of surgery may affect the risk of postoperative AKI. In a large prospective observational multi-center study [8], most (87.2%) of the postoperative AKIs developed after an open surgery. Among these, cardiac surgery had the highest risk of postoperative AKI. Similar to AKI in other hospitalized settings, cardiac-surgery-associated AKI has been associated with long-term risk of CKD, end-stage kidney disease, and mortality [11,12,13,14]. The aforementioned results suggest that postoperative AKI has been a significant healthcare burden worldwide.

Several risk factors for AKI after cardiac surgery [15,16], such as advanced age, female sex, hypertension, hyperlipidemia, diabetes, etc., have been reported previously in the literature. Both hypertension (odds ratio 1.770, 95% CI 1.291 to 2.427, *p* < 0.001) and diabetes (odds ratio 1.767, 95% CI 1.261 to 2.477, *p* = 0.001) have been reported as a risk factor for AKI in critically ill patients [2]. Since aging populations have become a common healthcare issue worldwide, the incidence of AKI in inpatients may continuously rise because of the increase in the prevalence of hypertension and diabetes associated with aging populations. Therefore, it is important to assess the risk of AKI and adopt appropriate actions to prevent it and improve patients’ outcomes. A recent study [17] proposed a perioperative laboratory test-based prediction model for AKI after cardiac surgery. Nevertheless, this model was developed based on data from a White-dominant population. The incidence of AKI was relatively high in some Asian regions [18], while there might be racial differences in terms of outcomes of AKI [19,20]. Furthermore, some factors (such as ethnicity and socioeconomic condition) may contribute to epidemiological variability in AKI worldwide, according to a recent review [18]. Hence, it is important to validate a prediction model in a different ethnic population. In this study, we examined the performance of the recently reported AKI prediction model [17] in a retrospective Asian cohort of patients undergoing cardiovascular surgery.

## 2. Materials and Methods

This was a retrospective study conducted in a tertiary medical center. The study protocol was approved by the ethics committee and the institutional review board of Taichung Veterans General Hospital, Taichung, Taiwan (approval number: CE19199B). The requirement of written informed consent was waived due to the retrospective study design, and de-identified data were used for analyses. We retrospectively identified patients who underwent elective cardiovascular surgery in our cardiovascular center between January 2017 and April 2019. We excluded those who underwent a surgical procedure other than coronary artery bypass graft, valve surgery, and aorta surgery [17], and those who underwent a percutaneous cardiovascular procedure (such as percutaneous aortic surgery). Aorta surgery included root, ascending, and thoracoabdominal aortic surgery. Valve surgery included aortic, mitral, pulmonary, and tricuspid valve surgery [17]. Patients who had preoperative AKI or CKD, defined as having a preoperative estimated glomerular filtration rate (eGFR) less than 60 mL/min/1.73 m^2^, were also excluded. To test the accuracy of a previous prediction model [17], patients who had missing laboratory data required for the prediction model were excluded from the analyses.

Patients’ eGFR was determined using the chronic kidney disease epidemiology collaboration (CKD-EPI) equation [21]. For women, eGFR = 144 × (0.993)^Age^ × (serum creatinine/0.7)^−0.329^, if serum creatinine ≤ 0.7 mg/dL; eGFR = 144 × (0.993)^Age^ × (serum creatinine/0.7)^−1.209^, if serum creatinine > 0.7 mg/dL. For men, eGFR = 141 × (0.993)^Age^ × (serum creatinine/0.9)^−0.411^, if serum creatinine ≤ 0.9 mg/dL; eGFR = 141 × (0.993)^Age^ × (serum creatinine/0.9)^−1.209^, if serum creatinine > 0.9 mg/dL. To test the accuracy of a previous prediction model [17], we used a modified Kidney Disease: Improving Global Outcomes (KDIGO) definition [22] for AKI, as was used in a previous study [17]. The definition of AKI is summarized in Table 1. Briefly, mild AKI was defined as an increase in serum creatinine by ≥0.3 mg/dL within 48 h after the surgery or an increase in serum creatinine to ≥1.5 times baseline within 7 days after the surgery [17,22]. Moderate to severe AKI was defined as serum creatinine increased by ≥2 times baseline or increased to ≥4 mg/dL within 72 h or 14 days after the surgery [17,22].

All patients received preoperative assessment by a board-certified anesthesiologist within 1 week prior to the surgery. For the assessment, patients’ demographic and laboratory data were reviewed, and their medical history was recorded. We collected our patients’ medical history and demographic and laboratory data from the electronic medical record after this study was approved by the ethics committee and the institutional review board. The required laboratory data for the prediction model [17] are summarized in Table 2. These included pre- and post-operative serum creatinine, albumin, potassium, sodium, bicarbonate, and blood urea nitrogen. In our hospital, follow-up of the metabolic panel after cardiovascular surgery is considered standard of care. The average time to test the metabolic penal was 4.2 h.

### Statistical Analysis

We conducted all statistical analyses using the Statistical Package for the Social Sciences (IBM SPSS version 22.0; International Business Machines Corp, New York, NY, USA). Continuous variables were reported as mean ± SD, while categorical variables were reported as number (percentage). Performance of the prediction model for AKI was assessed using the receiver operating characteristic (ROC) analysis and expressed as area under the ROC curve (AUC) [23]. To determine consistency of the performance of the prediction model, we examined the AUC of the prediction model in subgroups of age (<65 years and ≥65 years), sex (male and female), hypertension, and diabetes, all of which have been reported as risk factors for AKI following cardiac surgery [15]. A two-sided *p* value of less than 0.05 was considered statistically significant.

## 3. Results

**Figure 1:**
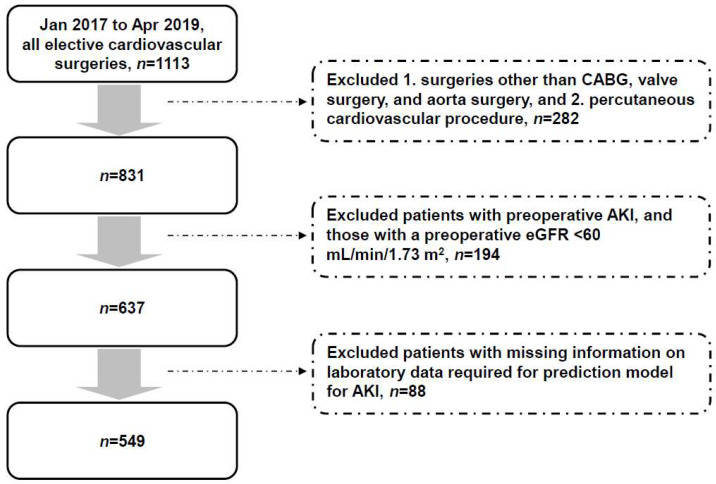
Flow diagram showing identification of the study cohort. AKI: acute kidney injury. eGFR: estimated glomerular filtration rate. CABG: coronary artery bypass graft.

Figure 1 shows the identification of the study cohort. We retrospectively identified 1113 patients who underwent cardiovascular surgery in our cardiovascular center between January 2017 and April 2019. After excluding those who did not meet the inclusion criteria and those with missing data, a total of 549 patients were analyzed. Table 3 shows the characteristics of these patients. The mean age was 61.6 ± 12.6 years, and most of the patients were men (70.3%). These patients had a medical history of hypertension (44.4%), coronary artery disease (26.4%), and diabetes (20.4%). The proportion of patients who underwent a coronary artery bypass graft, a valve surgery, and an aorta surgery were 35.0%, 35.9%, and 29.1%, respectively. The preoperative serum creatinine and eGFR were 0.89 ± 0.19 mg/dL and 84.9 ± 15.2 mL/min/1.73 m^2^, respectively. Cardiopulmonary bypass was performed in 313 (57%) of these patients during the surgery.

Table 4 shows the numbers of patients with AKI after the surgery, and the AUC of the prediction model. A total of 53 (9.7%) patients incurred mild AKI or worse after the surgery. This included 42 patients (7.7%) who had an increase in serum creatinine by ≥0.3 mg/dL within 48 h, and 40 patients (7.3%) who had an increase in serum creatinine to ≥1.5 times baseline within 7 days. The numbers of patients with moderate to severe AKI (an increase in serum creatinine by ≥2 times baseline or to ≥4 mg/dL) within 72 h and 14 days after the surgery were 13 (2.4%) and 19 (3.5%), respectively. The prediction model had a good predictive accuracy for AKI in our patients after the surgery (all AUC > 0.92, Table 4).

Table 5 shows the AUC of the prediction model in subgroups of age (<65 years and ≥65 years), sex (male and female), hypertension, and diabetes. The AUC in patients with age <65 years and ≥65 years were 0.946 (95% CI 0.898 to 0.994) and 0.910 (95% CI 0.832 to 0.987), respectively. Consistently, the AUC was higher than 0.85 with a *p* value < 0.001 in all the subgroups.

## 4. Discussion

In this study, we tested an AKI prediction model [17] derived using data from a non-Asian population in our patients who underwent cardiovascular surgery. The predictive accuracy was good for both mild and moderate to severe AKI after the surgery (Table 4). Furthermore, the prediction model works well in subgroups of age (<65 years and ≥65 years), sex (male and female), hypertension, and diabetes (Table 5). Our findings suggest that this model could be used to predict AKI risk following cardiac surgery in Asian patients. Identification of patients who are at risk for postoperative AKI and adoption of optimal preventive strategy may help improve patients’ outcomes.

AKI is a common complication in patients undergoing cardiovascular surgery and is associated with adverse long-term outcomes [15,16,24]. Most of the reported prediction models for AKI after cardiac surgery [17,25,26,27] are derived using data from non-Asian populations. Nevertheless, there might be ethnic disparities in the incidence and outcomes of postoperative AKI [19,20,28,29]. According to a large cohort study using data from the US Renal Data System [28], Asian patients had a lower likelihood of recovery of renal function (adjusted hazard ratio 0.82, 95% CI 0.69 to 0.96) after an episode of AKI compared with White patients. Moreover, genetic differences might be one of the factors that cause racial disparities in AKI after cardiac surgery. In a Southeast Asian population [29], genetic polymorphism of interleukin-6 was shown to be protective against the development of AKI after a cardiac surgery. This finding was contradictory to a previous study conducted in Caucasian patients [30], in which the genetic polymorphism of interleukin-6 was associated with a higher risk of renal dysfunction after cardiac surgery. These results suggest that population differences should be taken into account when developing predictive models for AKI after surgery. Hence, we tested the accuracy of a recently reported prediction model for AKI after cardiac surgery [17] in our patients with a preoperative eGFR ≥ 60 mL/min/1.73 m^2^. Our results suggest a good performance of the prediction model for mild to severe AKI in an Asian population who underwent major cardiovascular surgery (Table 4).

It is not yet clear whether the type of surgery may be associated with risk of postoperative AKI. According to a recent large observational multi-center study [8], most of the postoperative AKI developed after open surgery (1696 out of 1945, 87.2%). Among that cohort (*n* = 10,568) [8], a total of 1945 (18.4%) patients had a postoperative AKI. The proportion was similar in patients underwent open surgery (1696 out of 8954, 18.9%). The rate of postoperative AKI after robotic surgery was 21.5% (52 out of 242), although the case number was relatively low. The rate of AKI after robot-assisted laparoscopic prostatectomy was investigated in a recent meta-analysis [31]. The authors identified 10 studies with more than 60 thousand patients, and the pooled rate of AKI after robot-assisted laparoscopic prostatectomy was 7.2%. It might be reasonable that patients who underwent open surgery may have a higher risk of postoperative AKI, and we examined the prediction model in our patients who underwent open surgery. Nevertheless, further studies are needed to address the issues of AKI after robotic surgery, and the accuracy of a prediction model developed using data from open surgery in patients who underwent robotic surgery.

Postoperative AKI has been associated with the development of CKD and all-cause mortality independent of age, sex, body mass index, and some established risk factors [11,12,13,14,32,33]. Several risk factors (such as age, female sex, hypertension, diabetes, smoking history, etc.) for AKI following cardiac surgery have been reported, while multiple pathophysiological pathways (such as hypovolemia and ischemia, inflammation, vasoconstriction, oxidative stress, etc.) may be involved [15,16]. In a large cohort study [28] with more than 1 million patients analyzed, women (vs. men, adjusted hazard ratio 0.86, 95% CI 0.83 to 0.90, *p* < 0.001), hypertension (yes vs. no, adjusted hazard ratio 0.93, 95% CI 0.89 to 0.97, *p* < 0.001), and diabetes (yes vs. no, adjusted hazard ratio 0.91, 95% CI 0.87 to 0.95, *p* < 0.001) were associated with a lower likelihood of recovery of renal function after AKI. Similarly, critically ill patients with AKI were more likely to have hypertension (53.8% vs. 39.4%, *p* < 0.001) and diabetes (31.2% vs. 17.5%, *p* < 0.001) compared with those who did not have AKI in a multinational cross-sectional study [2]. In a multivariate adjusted model, hypertension and diabetes were both independently associated with AKI in critically ill patients. We examined the accuracy of the prediction model in our patients with or without established risk factors, such as age, female sex, hypertension, and diabetes (Table 5). Our findings suggest that the prediction model could be used in Asian patients who underwent a cardiac surgery independent of these risk factors.

Several pathophysiological pathways have been proposed to predispose patients to AKI following cardiac surgery. For example, hypoperfusion, atheroembolism, or vasoconstriction following activation of the sympathetic nervous system may lead to ischemia [15,16]. Hemolysis may cause the production of free radicals and reactive oxygen species. Inflammation and oxidative stress followed by activation of inflammatory mediators and complement may contribute to vasoconstriction and reduced renal perfusion [15,16]. Other factors, such as intraoperative and postoperative use of nephrotoxic agents, embolic factors, and genetic disposition, may also play a role in the development of postoperative AKI. However, there is no definitive prevention strategy for AKI following cardiovascular surgery despite the various interventions studied [15,16,24]. Since the development of AKI after cardiovascular surgery may be multifactorial, it is not surprising that previous singular interventions did not result in improvements in renal outcomes [24]. The effect of guideline-directed therapy on hospitalization for any causes was investigated in a recent pragmatic trial [34] for patients with CKD (defined as an eGFR less than 60 mL/min/1.73 m^2^ or the presence of proteinuria). More than 11 thousand patients with CKD, type 2 diabetes, and hypertension were randomly assigned to guideline-based interventions or usual care. Nevertheless, there was no significant difference in the rate of hospitalization for any causes (20.7% vs. 21.7%, *p* = 0.58) after 1 year. It is interesting to note that there was no significant between-group difference in the rate of AKI (12.7% vs. 11.3%). In contrast, applying care bundles to patients at high risk has been associated with a lower risk of AKI after cardiac surgery [35,36]. In a single-center randomized controlled trial [35], implementation of the KDIGO guidelines reduced the rate of AKI within 72 h (odds ratio 0.483, 95% CI 0.293 to 0.796, *p* = 0.004) compared with standard care. Although there was no significant difference in the risk of AKI within 72 h (odds ratio 1.21, 95% CI 0.76 to 1.95) in another multicenter randomized controlled trial [36] using the same method, the rate of moderate to severe AKI was reduced (odds ratio 0.52, 95% CI 0.28 to 0.96). In a large multicenter study [1], the effect of a clinical decision support system on AKI outcomes was investigated. The authors reported a significant reduction in hospital mortality risk (adjusted odds ratio 0.76, 95% CI 0.70 to 0.88, *p* < 0.001) and decrease in length of hospital stay (adjusted incidence rate ratio 0.91, 95% CI 0.89 to 0.92, *p* < 0.001). These benefits were observed in patients with AKI without affecting outcomes for patients without AKI. Hence, identification of patients at high risk for AKI following cardiac surgery and implementation of guidelines recommended care may help reduce the rate of AKI and subsequent adverse outcomes in these patients.

Nevertheless, more data are needed to support evidence-based interventions to improve long-term outcomes in patients with postoperative AKI, especially in Asian patients. It was reported that patients with AKI were at higher risk of incident or progressed CKD (hazard ratio 2.67, 95% CI 1.99 to 3.58), end-stage kidney disease (hazard ratio 4.81, 95% CI 3.04 to 7.62), and death (hazard ratio 1.80, 95% CI 1.61 to 2.02) in a systemic review and meta-analysis [5], in which 82 studies between 2004 and 2018 were included. Among the 82 studies, however, most were conducted in non-Asian regions with a retrospective study design. Only two studies were conducted in Asian patients who underwent a cardiac surgery with a prospective study design. Xu et al. [12] reported that patients with AKI after a cardiac surgery had a higher risk of all-cause mortality (adjusted hazard ratio 1.74, 95% CI 1.27 to 2.37, *p* = 0.001) during a follow-up period of 2 years. These patients also had a higher risk of incident progressive CKD (adjusted hazard ratio 20.32, 95% CI 4.55 to 97.31, *p* < 0.001) compared with those who did not have postoperative AKI. Similarly, Chew et al. [37] reported that patients with AKI after a cardiac surgery had a higher risk of incident end-stage kidney disease during a mean follow-up of 4.4 ± 2.8 years. The risk was even higher among patients who had stage 2 or stage 3 AKI (adjusted hazard ratio 5.8, 95% CI 1.769 to 18.732, *p* = 0.004), compared with those who had stage 1 AKI (adjusted hazard ratio 4.7, 95% CI 1.736 to 12.603, *p* = 0.002). The risk of 5-year all-cause mortality was significantly increased among these Asian patients who had AKI after a cardiac surgery. The adjusted hazard ratio were 1.7 (95% CI 1.165 to 2.571, *p* = 0.007) and 2.5 (95% CI 1.438 to 4.229, *p* < 0.001), respectively, for patients with stage 1 and stage 2–3 AKI. Since there are several emerging therapies for patients with CKD [38,39], further studies to address the issue of interventions to prevent postoperative AKI are required and expected.

There are several limitations in this study. Firstly, this was a retrospective study. There might be selection biases that have confounded our results. Secondly, we did not have detailed information on medications that might have effects on AKI (for example, nonsteroid anti-inflammatory drugs, renin-angiotensin-system blockers, and diuretics). Nevertheless, medications were not included in the AKI prediction model [17]. We considered application of the model to our patients reliable according to our results. Third, this was a single-center study with a relatively small sample size. Our findings need to be confirmed in future studies. Despite these limitations, our results suggest that AKI following cardiovascular surgery in our patients could be predicted using a model derived from a non-Asian population. Given that most of the previous studies were conducted in non-Asian populations [25,26,27], our findings are clinically relevant since the incidence of AKI is relatively high in some Asian regions [18].

## 5. Conclusions

In summary, we tested an AKI prediction model in our patients who underwent major cardiovascular surgery with a baseline eGFR ≥ 60 mL/min/1.73 m^2^. If confirmed in future studies, this model may be used to predict AKI in Asian patients after a cardiac surgery. Identification of high-risk patients and implementation of guidelines recommended care may help improve their outcomes.

## Figures and Tables

**Table 1:** Definition of acute kidney injury.

1. Mild: increase in sCr by ≥0.3 mg/dL within 48 h after surgery or increase in sCr to ≥1.5 times baseline within 7 days after surgery
2. Moderate to severe: sCr increase by ≥2 times baseline or increase to ≥4 mg/dL
A. Within 72 h after surgery
B. Within 14 days after surgery

sCr, serum creatinine.

**Table 2:** Variables required for the prediction model.

Preoperative serum creatinine
Perioperative change in serum creatinine
Postoperative serum albumin
Postoperative blood urea nitrogen
Postoperative serum potassium
Postoperative serum sodium
Postoperative serum bicarbonate
Duration in hours from end of surgery to postoperative metabolic panel

**Table 3:** Characteristics of the study patients on preoperative assessment.

Number of patients	549
Age, years	61.6 ± 12.6
Male, *n* (%)	386 (70.3)
Body mass index, kg/m^2^	24.9 ± 3.9
Systolic blood pressure, mm Hg	138 ± 24
Diastolic blood pressure, mm Hg	79 ± 14
Smoking, *n* (%)	93 (16.9)
Hypertension, *n* (%)	244 (44.4)
Coronary artery disease, *n* (%)	145 (26.4)
Diabetes, *n* (%)	112 (20.4)
Surgical procedure, *n* (%)	
Coronary artery bypass graft	192 (35.0)
Valve surgery	197 (35.9)
Aorta surgery	160 (29.1)
Preoperative serum creatinine, mg/dL	0.89 ± 0.19
Preoperative eGFR, mL/min/1.73 m^2^	84.9 ± 15.2

Values are mean ± SD or *n* (%). eGFR: estimated glomerular filtration rate.

**Table 4:** Numbers of patients with event and accuracy of the prediction model.

Acute Kidney Injury	Patients with Event, *n* (%)	Area under the ROC Curve
^a^ Mild	53 (9.7)	0.928 (95% CI 0.884 to 0.973)
^b^ Moderate to severe: within 72 h after surgery	13 (2.4)	0.999 (95% CI 0.997 to 1.000)
^b^ Moderate to severe: within 14 days after surgery	19 (3.5)	0.997 (95% CI 0.994 to 1.000)

^a^ Increase in serum creatinine by ≥0.3 mg/dL within 48 h after surgery or increase in serum creatinine to ≥1.5 times baseline within 7 days after surgery. ^b^ Serum creatinine increase by ≥2 times baseline or increase to ≥4 mg/dL. ROC: receiver operating characteristic.

**Table 5:** Accuracy of the prediction model for mild acute kidney injury in subgroups.

^a^ Mild Acute Kidney Injury	Area under the ROC Curve	*p*
Age < 65 years, *n* = 306	0.946 (95% CI 0.898 to 0.994)	<0.001
Age ≥ 65 years, *n* = 243	0.910 (95% CI 0.832 to 0.987)	<0.001
Male, *n* = 386	0.955 (95% CI 0.913 to 0.998)	<0.001
Female, *n* = 163	0.852 (95% CI 0.735 to 0.968)	<0.001
Hypertension (−), *n* = 305	0.941 (95% CI 0.894 to 0.987)	<0.001
Hypertension (+), *n* = 244	0.911 (95% CI 0.824 to 0.999)	<0.001
Diabetes (−), *n* = 437	0.920 (95% CI 0.867 to 0.972)	<0.001
Diabetes (+), *n* = 112	0.954 (95% CI 0.871 to 1.000)	<0.001

^a^ Increase in serum creatinine by ≥0.3 mg/dL within 48 h after surgery or increase in serum creatinine to ≥1.5 times baseline within 7 days after surgery. ROC: receiver operating characteristic.

## Data Availability

The data presented in this study are available on request from the corresponding author. The data are not publicly available due to privacy/ethical restrictions.
